# Apparent Total Tract Digestibility of Energy, Concentrations of Digestible Energy and Metabolizable Energy, and Nitrogen Balance in Growing Pigs Fed Bakery Meal and Biscuit Meal with Multi-Enzyme

**DOI:** 10.3390/ani15071002

**Published:** 2025-03-30

**Authors:** Jihwan Lee, Joeun Kim, Junseon Hong, Hyunju Park, Soojin Sa, Sungwoo Park, Yongmin Kim, Sungdae Lee, Yohan Choi, Yongdae Jeong

**Affiliations:** 1 Swine Science Division, National Institute of Animal Science, Rural Development Administration, Cheonan 31000, Republic of Korea; junenet123@naver.com (J.L.); kjektw@korea.kr (J.K.); gospel0342@korea.kr (J.H.); guswn707@korea.kr (H.P.); soojinsa@korea.kr (S.S.); woht3013@naver.com (S.P.); silveraz@korea.kr (Y.K.); 2 Precision Animal Nutrition Division, National Institute of Animal Science, Rural Development Administration, Wanju 55365, Republic of Korea; leesd@korea.kr

**Keywords:** apparent total tract digestibility, bakery meal, biscuit meal, nitrogen balance

## Abstract

A total of eight ([Landrace × Yorkshire] × Duroc) barrows with an initial body weight (BW) of 33.06 ± 1.16 kg were placed in metabolism crates. The dietary treatments were structured using a 4 × 2 factorial design, which included two factors: four diet types (i.e., corn-based diet, CON; corn-plain bread meal (PBM)-based diet, PBMD; corn-sweet bread meal (SBM)-based diet, SBMD; and corn-biscuit meal (BM)-based diet (BMD) with or without 0.1% multi-enzyme supplementation). In this study, the concentrations of DE and ME were 4075 and 3981 kcal/kg dry matter (DM) in two-source bakery meals (i.e., PBM and SBM) and 4153 and 4075 kcal/kg DM in BM. The DE and ME (on a DM basis) in the bakery meals (i.e., PBM and SBM) and BM were significantly higher (*p* < 0.05) than in corn. However, there was no significant difference (*p* > 0.05) in the ATTD of DM, GE, and N among the dietary treatments, multi-enzyme, and their interaction. In summary, the bakery meals and biscuit meals investigated in this study have the potential to partially replace corn. Further research is necessary to ascertain the effects of multi-enzyme supplements on bakery meals and biscuit meals.

## Simple Summary

Rising feed costs and climate change present significant challenges to the swine industry, prompting the exploration of alternative feed ingredients. Bakery meals and biscuit meals, initially produced for human consumption, have surfaced as viable substitutes for corn, mainly due to their rich energy and nutrient profiles. These meals provide higher energy levels than corn, and their nutrient compositions are also superior or comparable to those of corn. Despite the variability in nutrient composition that restricts its broader usage in pig feed, this study demonstrates that the nutrient content and digestibility of these meals are on par with corn, rendering them a promising alternative. Nonetheless, as the impacts of multi-enzyme supplements on food by-products have not been fully studied, additional research is warranted.

## 1. Introduction

Rising feed costs and climate change pose significant challenges to the sustainability of the swine industry. Feed costs account for about 70% of the overall production cost for swine [1,2]. Swine industry producers often face a significant increase in feed costs, which rose by approximately 12% in 2021, largely driven by increased feed imports following the recovery from African Swine Fever (ASF) in China [3,4]. Additionally, the Ukraine–Russia conflict has significantly disrupted the food supply chain, particularly affecting corn and wheat, which are primary feed sources for livestock. This disruption has led to a sharp increase in global grain prices [5]. The livestock industry contributes approximately 15% of the global greenhouse gas (GHG) emissions, significantly influencing global warming [6]. The production of 1 kg of pork contributes to the global warming potential (GWP), ranking second after beef, and is followed by chicken, eggs, and milk [7]. Consequently, these challenges have generated strong interest in utilizing unconventional feed ingredients and food by-products in the formulation of swine diets. Additionally, the global shift toward sustainable circular bio-economy strategies has led to a greater emphasis on recycling and repurposing various by-products that are edible but no longer suitable for human consumption [8]. In fact, by-products generated during food production have been utilized as key ingredients in swine diets. However, there is still a need for by-products that meet strict hygiene and safety standards [9]. Given that bakery and biscuit products are originally produced for human consumption, their by-products may serve as viable alternatives that meet these requirements. According to the previous literature, bakery meal and biscuit meal can serve as substantial sources of energy while providing protein and amino acids, highly comparable to those found in corn [10,11]. Bakery meal, a mixture of bread, cookies, cake, crackers, and dough, has also been considered as a feed ingredient for pigs [12,13]. Published data suggest that bakery meal is recognized for its good palatability and contains approximately 8 to 15% crude protein (CP), 5 to 10% ether extract (EE), and 3 to 10% ash [12,14,15]. Biscuit meal has gained attention as a feed ingredient for pigs due to its higher metabolizable energy (ME) than corn and its good palatability [16,17]. However, limited data are available regarding the nutritional value of biscuit meals, including energy, CP, EE, and other key nutrients. Additionally, the few studies available were only performed on poultry [18] and in vitro [19]. Although food by-products, including bakery meal and biscuit meal, offer sufficient nutritional value and good palatability compared to corn, they have not been widely used in pigs due to substantial variation in their nutritional composition, which depends on the diverse combinations of food ingredients. This limitation makes predicting nutrient utilization in pigs fed diets that include food by-products challenging. Only one recent study has determined net energy (NE), digestible energy (DE), and ME using 11 different sources of bakery meal [20]. Additionally, a single recent study has summarized the gross energy (GE), DE, and ME of biscuit meals [21]. However, there are no available data concerning the effects of adding multi-enzyme to bakery and biscuit meals, nor are there comparative data on energy digestibility among these ingredients along with corn. Thus, the primary objective of this study was to determine the apparent total tract digestibility (ATTD) of DE and ME in bakery and biscuit meals and to compare these values with those of corn. The secondary objective was to examine the effects of multi-enzyme on the ATTD of DE and ME in these ingredients.

## 2. Materials and Methods

### 2.1. Experimental Animal, Diets, Feeding and Design

Eight ([Landrace × Yorkshire] × Duroc) barrows with an initial body weight (BW) of 33.06 ± 1.16 kg were housed in metabolic cages (45 × 55 × 45 cm), each equipped with a feeder and a slatted floor for urine and feces collection. Corn and various food by-products (plain bread meal, PBM; sweet bread meal, SBM; and biscuit meal, BM) were acquired from local suppliers (DH Vital Feed, Pyeongtaek, Republic of Korea; Hanjungss, Seoul, Republic of Korea). The PBM was produced from dried plain white bread (e.g., sandwich bread) without filings or toppings, SBM was produced from sweet bread products containing filings (e.g., red bean, cream), and BM was produced from confectionery dough and related by-products, including cookies and crackers. These ingredients were transported to the National Institute of Animal Science where their nutrient composition was analyzed (Table 1). A corn-based basal diet (CON) and three experimental diets containing PBM (PBMD), SBM (SBMD), and BM (BMD) were developed (Table 2). Vitamins and minerals were added to all diets to satisfy or surpass the nutrient requirements outlined by the National Research Council (NRC) for pigs [22]. A multi-enzyme blend provided by Easy Bio (Seoul, Republic of Korea), composed of α-galactosidase, galactomannanase, xylanase, and β-glucanase, was included. The dietary treatments followed a 4 × 2 factorial design, examining four types of diets (CON, PBMD, SBMD, and BMD) both with and without 0.1% multi-enzyme supplementation. The research utilized an 8 × 8 Latin square design with eight dietary treatments. Pigs were weighed at the start of each period and fed thrice daily at an energy requirement for maintenance based on the individual BW of the pigs (197 kcal ME per kg BW^0.60^) [22]. The pigs received their feed at 0800 and 1700 h in two equal portions as dry mash. Each assessment period lasted 9 days, starting with 4 days of adaptation followed by 5 days dedicated to collecting fecal and urine samples. Feed leftover in the feeders was measured daily, and the feed intake (FI) was calculated by subtracting the residual feed from the total provided. Weigh-ins for the pigs were conducted at weaning (start of the experiment), before transfer to metabolism crates, and at the conclusion of both the adaptation and fecal collection periods.

### 2.2. Sample Collection and Chemical Analysis

Urine and fecal samples were collected over 4 days using the marker-to-marker approach [23]. The fecal collections commenced when chromic oxide, the first marker included in the morning meal on Day 5, was detected in the feces. This process continued until ferric oxide, the second marker included in the meal on Day 8, was observed in the feces. During this phase, fecal samples were collected twice daily and immediately stored at −20 °C, while urine was collected in buckets under the metabolism crates, containing 50 mL of 6N HCl as a preservative. The urine collection buckets were emptied daily, the total weight of the urine was recorded, and 20% of the urine was stored at −20 °C. At the conclusion of the experiment, stored fecal and urine samples were pooled according to individual animal and diet, and then divided into two subsamples. Fecal samples were dried in a 50 °C forced-air drying oven, and the urine samples were lyophilized. Fecal samples were ground through a 1 mm screen and thoroughly mixed prior to analysis. A sample from each feed ingredient, diet, feces, and urine was analyzed in duplicate. The components (corn, DBM, FBM, and BM), diets, ground fecal samples, and lyophilized urine samples were analyzed for GE using a bomb calorimeter (Model 6400, Parr Instruments, Moline, IL, USA). Additionally, the crude protein (CP) in the ingredients, diets, and fecal samples was determined using the Kjeltec TM 8400 (FOSS Inc., Eden Prairie, MN, USA), applying a conversion factor of 6.25 for CP estimation. Following chemical analysis, the ATTD of the GE, CP, and dry matter (DM) was calculated for each diet and also the DE and ME in each diet were determined [21]. The DE and ME contributions from corn were calculated by dividing the DE and ME of the corn diet by the inclusion rate of corn. These contributions were then subtracted from the DE and ME of the diets containing each food by-product, after which the DE and ME values for each food by-product were calculated by difference [23]. Nitrogen (N) digestibility, N retention, and N biological value were calculated using the method described by Hu et al. [24].

### 2.3. Statistical Analysis

A Shapiro–Wilk test and Levene’s test were used to assess normality and the equality of variances, respectively. All data were statistically analyzed using two-way analysis of variance (ANOVA) with JMP 16.0 (SAS Institute Inc., Cary, NC, USA), followed by Tukey’s honestly significant difference (HSD) test for post hoc comparisons. A *p*-value of less than 0.05 was considered to indicate a statistically significant difference and interaction.

## 3. Results

### 3.1. Energy Digestibility

There was no significant difference (*p* > 0.05) in feed intake (FI) across the treatments (Table 3). Relative to corn-based diets (CON), dry feces output was significantly higher (*p* < 0.05) in pigs fed BM diets (BMD), with the following order: BMD, SBM, and PBM. No significant differences (*p* > 0.05) were observed in the ATTD of DM across the dietary treatments. Pigs consuming BMD exhibited a higher (*p* < 0.05) gross energy (GE) intake compared to those on CON. Although not significant (*p* > 0.05), GE intake was numerically greater in pigs fed diets containing two sources of bakery meal (i.e., PBMD and SBMD) compared to those fed CON. Pigs on BMD also showed significantly higher (*p* < 0.05) fecal GE output compared to those on CON, and this output was further increased (*p* < 0.05) in pigs fed SBMD and PBMD. There were no significant differences (*p* > 0.05) in urine GE output among the dietary treatments. The ATTD of GE was not affected (*p* > 0.05) by the dietary treatments. Additionally, multi-enzyme supplementation did not influence (*p* > 0.05) FI, GE intake, dry feces output, or GE output in feces and urine, resulting in no differences in ATTD of DM and GE. Moreover, no significant interaction (*p* > 0.05) was found between dietary treatments and enzyme supplementation.

Compared to CON, the concentration of DE and ME in BMD was the highest (*p* < 0.0001), followed by SBMD and PBMD (Table 4). The concentrations of DE and ME in BM (as-fed basis and DM basis) were greater (*p* < 0.0001) than those in other ingredients. Likewise, the concentrations of DE and ME in SBM and PBM (as-fed basis) were higher than those in corn. However, there was no significant difference (*p* > 0.05) in the concentration of DE and ME (DM basis) between two-source bakery meals (i.e., SBM and PBM) and corn.

### 3.2. Nitrogen Balance

The nitrogen (N) intake in pigs fed two-source bakery meal diets (i.e., PBMD and SBMD) was greater (*p* < 0.0001) than in pigs fed CON and BMD (Table 5). There was no significant difference (*p* > 0.05) in N excretion in feces and urine, ATTD of N, N retention (%), and biological value among dietary treatments. Likewise, multi-enzyme supplementation did not affect (*p* > 0.05) N balance including the ATTD of N and biological value. However, N retention (%) tended (*p* = 0.0639) to increase with multi-enzyme supplementation.

## 4. Discussion

Various alternative ingredients have been utilized as substitutes for corn to ensure that animals’ dietary nutrient needs are met through appropriate formulation [25]. The accurate estimation of nutrient concentrations, including energy, in alternative ingredients is essential for formulating balanced diets that satisfy the nutritional requirements of animals [26]. Among them, food by-products such as bakery meals and biscuit meals have emerged as promising options to reduce grain usage and mitigate negative environmental impacts, garnering significant attention [27]. In this study, the analyzed concentration of DM and GE in two sources of bakery meal was inconsistent, whereas the concentration of CP was consistent with previously reported values [22]. For example, the concentration of DM and GE in plain bread meal (PBM) (DM: 87.5%, GE: 4001 kcal/kg) and sweet bread meal (SBM) (DM: 88.1%, GE: 4231 kcal/kg) was lower than that reported by NRC (DM: 90.8%, GE: 4558 kcal/kg). The concentration of CP in PBM (12.1%) and SBM (12.3%) was comparable to that reported by NRC (12.3%). However, a limitation of the NRC data is that it is based on a single source of bakery meals. The results for nutrient concentration in two sources of bakery meal aligned with values reported in studies conducted over the past 12 years [14,20,28,29]. Previous studies indicated that the concentration of DM in bakery meals ranged from 84.9% to 90.2%, and our values fell within this range. These studies also reported that concentrations of GE and CP ranged from 3687 kcal/kg to 4225 kcal/kg and from 7.46% to 13.50%, respectively. Likewise, the concentrations of GE and CP in our two sources of bakery meal fell within these ranges. However, there was some variation in the nutrient composition among the sources of bakery meal used in previous studies and this study. This variation is likely due to the sequence of mixing various products during the production of bakery meals. As for the biscuit meal, the nutrient composition was inconsistent with previous studies [16,30]. The DM content (88.9%) in our biscuit meal was less than that in biscuit meals used in previous studies (ranging from 89.3% to 92.4%, typically 91.0%). Moreover, the concentration of CP (8.01%) in our biscuit meal was lower than the values reported by Manu et al. [31] and Ojediran et al. [13] (9.9% and 9.4%, respectively). However, the concentration of CP in the biscuit meal used in this study was consistent with a previously reported value [32]. Similar to bakery meals, the nutrient composition of biscuit meals also showed some variation, likely due to differences in the types and proportions of products used, including crackers and dough. The nutrient composition, including the amino acids in two-source bakery meal and biscuit meal used in this study, was found to be quite comparable to or higher than that of corn, the primary energy source. Therefore, considering the nutrient content, it can be concluded that these ingredients are not deficient in comparison to corn. In this study, a corn-based diet was used to specifically evaluate the substitution of the energy component with bakery and biscuit meal. Further research may consider these by-products as a partial replacement for both energy and protein sources in a corn-soybean meal-based diet.

The higher GE intake for pigs fed two-source bakery meals diet (i.e., PBMD and SBMD) and biscuit meal diet (BMD) compared to those on corn diets (CON) might result from the fact that the GE of food by-products is typically higher than that of corn. Supporting this assertion, DE and ME (on both as-fed and DM basis) for three food by-products were greater than those for corn. Additionally, pigs fed diets of three food by-products tended to have less leftover feed. According to prior studies, bakery meals and biscuit meals demonstrated superior palatability [15,17]. Consequently, their high palatability, combined with inherent high energy contents, might contribute to the increased GE intake of diets based on these three food by-products compared to corn diets. In this study, no significant differences were noted in the ATTD of DM and GE among dietary treatments. This result might be attributable to increased outputs of dry feces, fecal GE, and urine GE in pigs fed diets of three food by-products. Typically, the ATTD of DM in corn is about 88% [33,34], but it was observed to be around 90% in this study. Although this difference is minimal, it might be due to varying ingredient quality as nutrient composition can differ depending on harvest timing and processing methods. Stein et al. [20] found that the average ATTD of GE across 11 bakery meal sources was approximately 83%, with average DM and ME values of 3951 and 3655 kcal/kg DM, respectively. Notably, DE and ME ranged from 3607 to 4172 kcal/kg DM and 3450 to 4004 kcal/kg DM among these sources. In this study, the ATTD of GE in bakery meals was approximately 86%, with DM and ME values of 4075 and 3981 kcal/kg DM, respectively, fitting within the ranges reported by Stein et al. [20]. In the case of biscuit meal, no studies have yet analyzed its digestibility and inherent energy value using either direct or indirect methods. Here, the DM and ME values for the biscuit meal were recorded at 4153 and 4075 kcal/kg DM. Bakery and biscuit meals are generally comprised of a mix of cereal flour and cereal co-products, and sometimes include whole grains to lower moisture content before their use as livestock feed. Furthermore, during the production of bakery meals, cereal co-products or soybean meal may be added to processed food items to meet certain nutritional standards. Although this practice contributes to nutrient composition variability in food by-products, it also demonstrates the potential to develop cost-effective and nutritionally viable alternative feed ingredients. This was evident in this study, where bakery meal was identified as a more affordable yet effective alternative to corn. The higher N intake for pigs fed two-source bakery meals compared to those fed corn diets and biscuit meal diets reflects the fact that the CP content in two-source bakery meals is higher than in corn and biscuit meals. More specifically, the CP of the bakery meals used in this study was approximately 12%, whereas corn and biscuit meal had CP levels of approximately 7.9% and 8.0%, respectively. The CP contents in the bakery meals were consistent with findings from previous studies [29,35]. Consequently, their naturally high CP contents may contribute to increased N intake and N retention (g/d) compared to the diets containing corn and biscuit. In this study, there was no significant difference in the ATTD of DM and GE among the dietary treatments. Luciano et al. [36] reported that while the ATTD of DM of weaning pigs fed a diet containing salty bakery products was similar to those fed a wheat-based diet, the ATTD of DM of weaning pigs fed a diet containing sweet confectionery products was decreased compared with those fed a diet containing salty bakery products and wheat-based diet. In addition, Mazzoleni et al. [37] reported that the ATTD of GE in growing and finishing pigs fed diets containing salty bakery and sweet confectionery products was improved compared to the control diets. They suggested that discrepancy results from digestibility may be due to the type of carbohydrates, differences in starch processing, and tannins derived from sugary ingredients (e.g., chocolate products).

Moreover, no significant differences were observed in the ATTD of N, N retention (%), and biological values among the dietary treatments. These outcomes may be attributed to an increased N output, although no statistical difference was evident. However, Mazzoleni et al. [37] reported that the ATTD of CP in growing pigs fed diets containing salty bakery products was improved compared to the control diets. They attributed this improvement to the lower fiber content of the salty bakery products. When the proximate composition of the ingredients was analyzed prior to the commencement of the experiment, it was observed that the bakery meal used had a higher NDF than corn. This difference in ingredient composition may explain the contrasting results between the two studies. NDF consists of cellulose, hemicellulose, and lignin, and ADF consists of cellulose and lignin. NSPase has been known to enhance the breakdown of fibrous components such as NDF, IDF, TDF, and cellulose, and insoluble hemicellulose may facilitate a greater release and absorption of monosaccharides, thereby contributing to increased energy availability for pigs. Thus, we hypothesized that supplementation with a multi-enzyme (NSPase) could further enhance nutrient utilization of food by-products because the fiber content can influence the ATTD of nutrients and energy [38]. However, it is known that bakery meal has a lower fiber content compared to cereal grains [39,40]. This disparity likely results from the fact that cereal grains might be incorporated into food by-product procedures to meet specific nutritional standards. In this study, significant differences in energy and nitrogen digestibility with or without the addition of multi-enzyme were not observed. Consistent with our findings, Kiarie et al. [41], Song et al. [42], and Oladele et al. [43] reported no significant differences in energy, nitrogen, and amino acid digestibility when supplementing an enzyme mixture in cereal grains. On the other hand, numerous studies have demonstrated that multi-enzyme supplementation in diets improves nutrient digestibility [44,45,46]. The impact of multi-enzymes on nutrient digestibility does not always yield consistent results, as the efficacy of exogenous enzymes can be influenced by both their enzymatic activity and the availability of suitable substrates. Additionally, the aforementioned studies focused on the addition of exogenous enzymes to cereal grain-based diets. One limitation of this study is the use of a single enzyme dose. Therefore, further research is necessary to evaluate multiple levels of enzyme supplementation in order to determine the optimal dose and to better understand the interaction between exogenous enzymes and food by-products.

## 5. Conclusions

Two sources of bakery meals and biscuit meals have a comparable ATTD of GE and N to corn, and they also contain greater DE, ME, and CP. These results suggest that the bakery meals and biscuit meals evaluated in this study could be partially substituted for corn. However, the addition of multi-enzyme did not significantly affect nutrient utilization when supplemented in the food by-products (i.e., bakery meals and biscuit meals). Therefore, further research is necessary to assess the impacts of multi-enzyme supplements on bakery meals and biscuit meals.

## Figures and Tables

**Table 1:** Nutrient composition of by-product used in this study ^1^.

Items, %	Corn	PBM	SBM	BM
DM	84.3	87.5	88.1	88.9
GE, kcal/kg	3879	4001	4231	4829
CP	7.97	12.10	12.30	8.01
Ash	1.14	3.59	2.31	4.06
EE	3.34	4.02	6.62	10.62
NDF	9.30	16.24	12.24	5.50
ADF	3.10	5.93	7.62	2.20
Indispensable AA				
Arg	0.35	0.70	0.58	0.31
His	0.22	0.34	0.28	0.14
Ile	0.25	0.58	0.48	0.23
Leu	0.99	1.07	0.89	0.47
Lys	0.21	0.58	0.48	0.18
Met	0.16	0.22	0.18	0.11
Phe	0.35	0.68	0.57	0.32
Thr	0.27	0.50	0.42	0.19
Trp	0.06	0.14	0.12	0.06
Val	0.35	0.71	0.59	0.27
Dispensable AA				
Ala	0.53	0.71	0.59	0.44
Asp	0.58	0.97	0.81	0.63
Cys	0.16	0.29	0.24	0.15
Glu	1.26	2.21	1.84	2.36
Gly	2.91	0.61	0.51	0.26
Pro	0.65	0.86	0.72	0.68
Ser	0.37	0.54	0.45	0.29
Tyr	0.24	0.53	0.44	0.31
Total AA	9.91	12.23	10.19	7.40

^1^ Abbreviation: Corn, corn; PBM, plain bread meal; SBM, sweet bread meal; BM, biscuit meal; GE, gross energy; DM, dry matter; CP, crude protein; EE, ether extract; NDF, neutral detergent fiber; ADF, acid detergent fiber; Arg, arginine; His, histidine; Ile, isoleucine; Leu, leucine; Lys, lysine; Met, methionine; Phe, phenylalanine; Thr, threonine; Trp, tryptophan; Val, valine; Ala, alanine; Asp, aspartic acid; Cys, cystine; Glu, glutamic acid; Gly, glycine; Pro, proline; Ser, serine; Tyr, tyrosine.

**Table 2:** Ingredients and composition of the experimental diets ^1^.

Items, %	CON	PBMD	SBMD	BMD	Corn	PBMD	SBMD	BMD
	−				+			
Corn	97.00	47.00	47.00	47.00	97.00	47.00	47.00	47.00
By-product	-	50.00	50.00	50.00	-	50.00	50.00	50.00
Di-calcium phosphate	1.50	1.50	1.50	1.50	1.48	1.48	1.48	1.48
Limestone	0.80	0.80	0.80	0.80	0.75	0.75	0.75	0.75
Salt	0.40	0.40	0.40	0.40	0.37	0.37	0.37	0.37
Vitamin-mineral premix ^2^	0.30	0.30	0.30	0.30	0.30	0.30	0.30	0.30
Multi-enzyme	-	-	-	-	0.1	0.1	0.1	0.1
Total	100	100	100	100	100	100	100	100
Analyzed value								
DM, %	84.99	83.89	82.48	84.01	84.99	83.88	82.50	84.03
GE, kcal/kg	3818	3879	3951	4052	3818	3879	3951	4052
CP	7.68	9.73	9.82	7.91	7.68	9.74	9.80	7.92
EE	4.03	4.24	3.47	5.32	4.05	4.26	3.42	5.34
NDF	8.93	12.40	11.42	6.32	8.93	12.48	11.40	6.33
ADF	2.98	4.39	4.82	1.89	2.99	4.36	4.84	1.85

^1^ Abbreviation: CON, corn-based diet; PBMD, corn-plain bread meal-based diet; SBM, corn-sweet bread meal-based diet; BMD, corn-biscuit meal-based diet; −, dietary treatments without multi-enzyme supplementation; +, dietary treatments with 0.1% multi-enzyme supplementation; GE, gross energy; DM, dry matter; CP, crude protein; NDF, neutral detergent fiber; ADF, acid detergent fiber; EE, ether extract. ^2^ vitamin A as retinyl acetate, 10,622 IU; vitamin D3 as cholecalciferol, 1660 IU; vitamin E as DL-alpha-tocopheryl acetate, 66 IU; vitamin K as menadione nicotinamide bisulfate, 1.40 mg; thiamin as thiamine mononitrate, 1.08 mg; riboflavin, 6.49 mg; pyridoxine as pyridoxine hydrochloride, 0.98 mg; vitamin B12, 0.03 mg; D-pantothenic acid as D-calcium pantothenate, 23.2 mg; niacin, 43.4 mg; folic acid, 1.56 mg; biotin, 0.44 mg; Cu, 20 mg as copper chloride; Fe, 123 mg as iron sulfate; I, 1.24 mg as ethylenediamine dihydriodide; Mn, 59.4 mg as manganese hydroxychloride; Se, 0.27 mg as sodium selenite and selenium yeast; Zn, 124.7 mg as zinc hydroxychloride.

**Table 3:** Effects of various dietary ingredients and the supplementation of multi-enzymes on the apparent total tract digestibility (ATTD) of dry matter (DM) and gross energy (GE) in experimental diets provided to pigs ^1^.

Diet	Enzyme	Intake		Fecal Excretion			Urine Excretion			Digestibility	
		Feed Intake, g/d	GE Intake, kcal/d	Dry Feces Output, g/d	GE in Feces, kcal/kg	Fecal GE Output, kcal/d	Urine Output, g/d	GE in Urine, kcal/kg	Urine GE Output, kcal/d	ATTD of DM, %	ATTD of GE, %
CON	−	1029.83	3931.90	112.78	4574.61	515.49	1827.50	46.10	83.00	90.39	86.89
PBMD	−	1083.17	4201.60	127.22	4493.99	570.94	2170.00	41.08	87.77	90.67	86.39
SBMD	−	1088.00	4298.69	128.33	4580.95	588.08	2258.75	40.27	92.09	90.86	86.22
BMD	−	1106.00	4481.51	136.11	4586.84	624.39	1740.42	42.01	73.11	89.62	85.95
CON	+	1092.50	4171.17	117.78	4537.45	534.97	1721.25	46.91	78.28	91.06	87.05
PBMD	+	1101.33	4272.07	126.11	4637.09	585.41	1969.17	39.37	78.06	89.41	86.30
SBMD	+	1103.22	4358.81	133.33	4577.32	610.11	1907.92	40.81	79.93	89.63	85.96
BMD	+	1113.00	4509.88	135.00	4679.02	631.99	1783.33	41.66	72.67	90.43	85.98
SEM		37.592	147.95	3.303	57.320	17.174	157.938	3.337	8.674	0.656	0.478
Main factor											
Diet											
CON		1061.17	4051.53 ^b^	115.28 ^c^	4556.03	525.23 ^c^	1774.38	46.51	80.64	90.73	86.97
PBMD		1092.25	4236.84 ^ab^	126.67 ^b^	4565.54	578.17 ^b^	2069.58	40.23	82.91	90.04	86.35
SBMD		1095.61	4328.75 ^ab^	130.83 ^ab^	4632.93	599.10 ^ab^	2083.33	40.54	86.01	90.25	86.09
BMD		1109.50	4495.69 ^a^	135.56 ^a^	4579.13	628.19 ^a^	1761.88	41.84	72.89	90.03	85.96
SEM		26.581	104.616	2.336	40.531	12.144	111.679	2.359	6.134	0.464	0.338
	Enzyme										
	−	1076.75	4228.43	126.11	4559.10	574.72	1999.17	42.36	83.99	90.39	86.36
	+	1102.51	4327.98	128.06	4607.72	590.62	1845.42	42.19	77.23	90.13	86.32
	SEM	18.796	73.975	1.652	28.660	8.587	78.969	1.668	4.337	0.328	0.239
*p*-value											
Diet		0.6261	0.0359	<0.0001	0.5470	<0.0001	0.0696	0.2248	0.4835	0.6860	0.1733
Enzyme		0.3383	0.3470	0.4101	0.2373	0.1980	0.1763	0.9411	0.2769	0.5888	0.9109
Diet × Enzyme		0.8805	0.8920	0.6377	0.3765	0.9764	0.6525	0.9813	0.9084	0.2208	0.9766

^1^ Abbreviation: CON, corn-based diet; PBMD, corn-plain bread meal-based diet; SBM, corn-sweet bread meal-based diet; BMD, corn-biscuit meal-based diet; SEM, standard error of means; −, dietary treatments without multi-enzyme supplementation; +, dietary treatments with 0.1% multi-enzyme supplementation. ^a,b,c^ Means within a row with different superscript letters indicate significant differences at *p* < 0.05.

**Table 4:** Effects of different dietary ingredients and supplementation with multi-enzyme on the concentration of digestible energy (DE) and metabolizable energy (ME) in experimental diets fed to pigs ^1^.

Diet	Energy Content in Experimental Diets, kcal/kg		Ingredient	Energy Content in Feed Ingredients, kcal/kg			
	DE	ME		As-Fed Basis		DM Basis	
				DE	ME	DE	ME
CON	3320.43 ^c^	3243.75 ^c^	Corn	3440.97 ^d^	3361.52 ^c^	4081.81 ^b^	3987.57 ^b^
PBMD	3349.41 ^c^	3273.55 ^bc^	PBM	3550.38 ^c^	3469.96 ^b^	4057.57 ^b^	3965.67 ^b^
SBMD	3401.37 ^b^	3321.77 ^b^	SBM	3605.46 ^b^	3521.08 ^b^	4092.46 ^ab^	3996.69 ^b^
BMD	3483.28 ^a^	3417.20 ^a^	BM	3692.28 ^a^	3622.23 ^a^	4153.29 ^a^	4074.50 ^a^
SEM	13.254	15.731	SEM	13.955	16.589	16.069	19.023
*p*-value	<0.0001	<0.0001	*p*-value	<0.0001	<0.0001	0.0012	0.0014

^1^ Abbreviation: Corn, corn; PBM, plain bread meal; SBM, sweet bread meal; BM, biscuit meal; CON, corn-based diet; PBMD, corn-plain bread meal-based diet; SBM, corn-sweet bread meal-based diet; BMD, corn-biscuit meal-based diet; SEM, standard error of means. ^a,b,c,d^ means within a row with different superscript letters are significantly different at *p* < 0.05.

**Table 5:** Effects of various dietary ingredients and multi-enzyme supplementation on nitrogen (N) balance and apparent total tract digestibility (ATTD) of N in experimental diets fed to pigs ^1^.

Diet	Enzyme	N Intake, g/d	N Excretion in Feces, g/d	N Excretion in Urine, g/d	ATTD of N, %	N Retention, g/d	N Retention, %	Biological Value ^2^
CON	−	12.65	2.50	1.56	80.30	8.60	67.85	84.46
PBMD	−	16.86	3.04	1.86	81.96	11.86	70.53	86.07
SBMD	−	17.09	3.36	1.90	80.31	11.84	69.08	86.01
BMD	−	14.00	2.93	1.45	78.82	9.61	68.29	86.54
CON	+	13.42	2.59	1.49	80.60	9.34	69.54	86.12
PBMD	+	16.86	2.81	1.65	83.53	12.69	73.94	88.57
SBMD	+	17.33	2.91	1.61	83.23	12.82	73.91	88.79
BMD	+	14.09	2.83	1.53	79.91	9.73	69.07	86.36
SEM		0.529	0.217	0.193	1.433	0.527	1.987	1.532
Main Factor								
Diet								
CON		13.04 ^b^	2.54	1.53	80.45	8.97 ^b^	68.70	85.29
PBMD		17.00 ^a^	2.92	1.80	82.74	12.28 ^a^	72.24	87.32
SBMD		17.21 ^a^	3.13	1.75	81.77	12.33 ^a^	68.68	87.40
BMD		14.04 ^b^	2.88	1.49	79.37	9.67 ^b^	71.49	86.45
SEM		0.374	0.153	0.136	1.013	0.373	1.405	1.083
	Enzyme							
	−	15.15	2.96	1.72	80.35	10.48	68.94	85.77
	+	15.50	2.78	1.57	81.82	11.15	71.62	87.46
	SEM	0.264	0.108	0.096	0.717	0.263	0.993	0.766
*p*-value								
Diet		<0.0001	0.1724	0.2714	0.1115	<0.0001	0.1721	0.4903
Enzyme		0.3613	0.2685	0.2740	0.1547	0.0804	0.0639	0.1261
Diet × Enzyme		0.9252	0.6441	0.7161	0.8283	0.8568	0.7466	0.7691

^1^ Abbreviation: CON, corn-based diet; PBMD, corn-plain bread meal-based diet; SBM, corn-sweet bread meal-based diet; BMD, corn-biscuit meal-based diet; SEM, standard error of means; −, dietary treatments without multi-enzyme supplementation; +, dietary treatments with 0.1% multi-enzyme supplementation. ^2^ Biological value was calculated as follows: [N retention/(N intake − N excretion in feces)] × 100. ^a,b^ means within a row with different letters are significantly different at *p* < 0.05.

## Data Availability

The raw data supporting the conclusions of this article will be made available by the authors on request.

## Abbreviations

The following abbreviations are used in this manuscript:

ASF	African Swine Fever
ADF	Acid detergent fiber
ANOVA	Analysis of variance
ATTD	Apparent total tract digestibility
BM	Biscuit meal
BW	Body weight
CP	Crude protein
PBM	Plain bread meal
DE	Digestible energy
DM	Dry matter
EE	Ether extract
SBM	Sweet bread meal
FI	Feed intake
GE	Gross energy
GHS	Greenhouse gas
GWP	Global warming potential
HSD	Honestly significant difference
ME	Metabolizable energy
N	Nitrogen
NDF	Neutral detergent fiber
NE	Net energy
NRC	National Research Council
SEM	Standard error of means
